# Polycomb protein EZH2 suppresses apoptosis by silencing the proapoptotic miR-31

**DOI:** 10.1038/cddis.2014.454

**Published:** 2014-10-23

**Authors:** Q Zhang, S K R Padi, D J Tindall, B Guo

**Affiliations:** 1 Department of Pharmaceutical Sciences, College of Pharmacy, North Dakota State University, Fargo, ND 58108, USA; 2 Department of Urology, Biochemistry and Molecular Biology, Mayo Clinic College of Medicine, Rochester, MN 55905, USA

## Abstract

Enhancer of zeste homolog 2 (EZH2) is the catalytic subunit of the polycomb repressive complex 2 and suppresses gene expression by catalyzing histone H3 methylation on lysine 27. EZH2 is overexpressed in metastatic prostate cancer and has been shown to promote cell proliferation and metastasis. Here we show that EZH2 also suppresses prostate cancer apoptosis by coordinating the epigenetic silencing of two proapoptotic microRNAs (miRNA), miR-205 and miR-31. We previously reported that miR-205 promotes apoptosis by targeting antiapoptotic protein Bcl-w and miR-205 is silenced in prostate cancer through promoter methylation. In this study, we found that EZH2 suppresses miR-31 expression by trimethylation of lysine 27 on histone 3 on the miR-31 promoter. SiRNA knockdown of EZH2 increased miR-31 expression and decreased the antiapoptotic protein E2F6 (E2F transcription factor 6) (a target of miR-31), resulting in the sensitization of prostate cancer cells to docetaxel-induced apoptosis. Conversely, overexpression of EZH2 blocked docetaxel-induced apoptosis. We further demonstrated that miR-205 silencing is linked to miR-31 silencing through EZH2. Suppression of miR-205 with an miRNA inhibitor caused an increase of EZH2 protein, which in turn inhibited miR-31 expression. Conversely, overexpression of miR-205 decreased EZH2 protein and increased miR-31 expression. In paired human prostate cancer specimens and adjacent normal tissues, we observed that the decrease of miR-205 expression correlated with EZH2 overexpression and miR-31 silencing. Thus, EZH2 integrates the epigenetic silencing of miR-205 and miR-31 to confer resistance to chemotherapy-induced apoptosis.

Posttranslational modifications of histones (such as acetylation or methylation on the lysine residues) have important roles in regulating gene expression. Enhancer of zeste homolog 2 (EZH2) is the catalytic subunit of the polycomb repressive complex 2 (PRC2).^1^ It suppresses gene expression by catalyzing the methylation of lysine 27 on histone 3 (H3K27).^1^ PRC2 can catalyze sequential methylation at H3K27, producing mono-, di-, and trimethylated H3K27 (H3K27me1, H3K27me2, and H3K27me3).^2^ The PRC2 complex contains five subunits, including EZH2, EED, SUZ12, RbAp46/48, and AEBP2. EZH2 is overexpressed in a variety of cancers including prostate cancer.^3^ In prostate cancer, EZH2 is the most significantly upregulated gene found in metastasis.^4^ High levels of EZH2 expression correlates with prostate cancer progression and predicts a higher risk of recurrence after radical prostatectomy.^4,5^ The oncogenic activity of EZH2 has been extensively studied and most results are focusing on its function in promoting proliferation and metastasis.^6, 7, 8, 9, 10^ Recent studies have also demonstrated that EZH2 can suppress apoptosis.^11, 12, 13, 14, 15, 16, 17^ However, the mechanisms of apoptosis suppression by EZH2 remain poorly understood.

MicroRNAs (miRNAs) are small noncoding RNAs with a length of about 22 nucleotides and regulate gene expression by translational repression or inducing degradation of the target mRNAs.^18^ Dysregulation of various miRNAs has been shown to have critical roles in cancer biology, ranging from proliferation,^19^ differentiation,^20,21^ metabolism,^22^ metastasis,^23^ to apoptosis.^24^ We have recently reported that miR-205 and miR-31 are significantly downregulated in cell lines representing advanced prostate cancer.^25^ As a result, the expression of two antiapoptotic genes, miR-205 target *BCL2L2* (encoding Bcl-w) and miR-31 target *E2F6* (E2F transcription factor 6), is increased to contribute to resistance to apoptosis.^25^ Although we identified DNA promoter hypermethylation as a mechanism for miR-205 silencing,^25^ we did not know how miR-31 is suppressed in prostate cancer. Recently, EZH2-mediated histone methylation was shown to suppress miR-31 expression in adult T-cell leukemia.^26^ In this report, we show that EZH2 silences miR-31 in prostate cancer by catalyzing H3K27 trimethylation on the miR-31 promoter. Furthermore, we show that EZH2 contributes to apoptosis resistance by integrating DNA methylation-mediated miR-205 silencing with histone methylation-mediated silencing of miR-31. These findings provide a new mechanism for EZH2 suppression of apoptosis.

## Results

### EZH2 suppresses miR-31 expression

**Figure 1:**
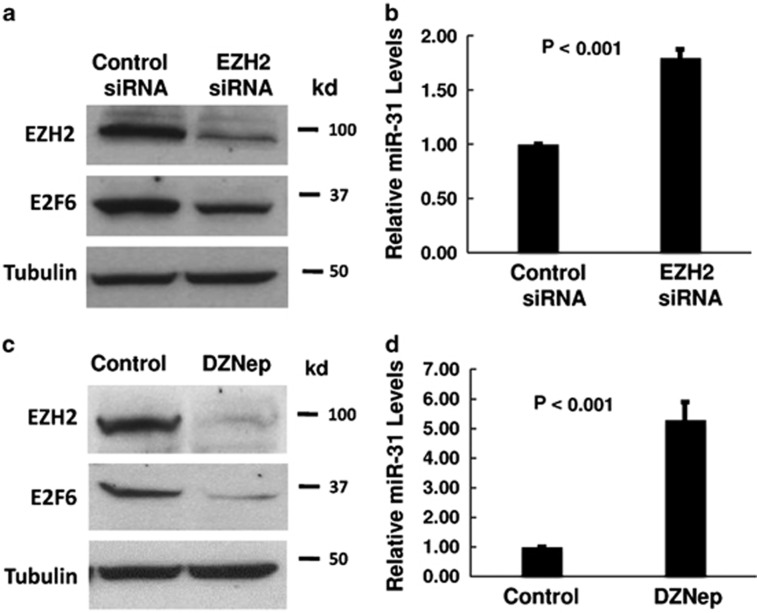
EZH2 suppresses miR-31 expression. (**a** and **b**) PC-3 cells were transfected with the negative control or EZH2 targeting siRNAs for 48 h. (**a**) Cell lysates were analyzed by western blot using the indicated antibodies. (**b**) Total RNA was isolated from the cells and real-time PCR analysis was performed as described in Materials and Methods. (**c** and **d**) PC-3 cells were treated with 5 *μ*M of DZNep for 24 h. (**c**) Cell lysates were analyzed by western blot using the indicated antibodies. (**d**) Total RNA was isolated from the cells and real-time PCR analysis was performed. All experiments have been repeated three times, and data shown are mean values±S.D.

We reported previously that miR-31 expression was decreased in prostate cancer cells, resulting in resistance to apoptosis.^25^ It was recently shown that in adult T-cell leukemia,^26^ the PRC can be recruited to the miR-31 promoter (on chromosome 9q21) by transcription factor YY1. Subsequently, EZH2 increases trimethylated H3K27 and suppresses miR-31 expression.^26^ To understand the mechanism of miR-31 silencing in prostate cancer, we examined if miR-31 is suppressed by EZH2. We found that siRNA knockdown of EZH2 restored miR-31 expression in PC-3 cells (Figures 1a and b). As a result, the antiapoptotic protein E2F6 (a target of miR-31^25^) was decreased by EZH2 siRNA treatment. Similarly, depletion of EZH2 with DZNeP (a known EZH2 inhibitor^27^) increased miR-31 expression in PC-3 cells and decreased E2F6 (Figures 1c and d). In another prostate cancer cell line DU-145, we also observed that siRNA knockdown of EZH2 or DZNeP treatment increased miR-31 expression and decreased E2F6 (Supplementary Figures 1A and B).

### EZH2 regulates histone methylation on the miR-31 promoter

**Figure 2:**
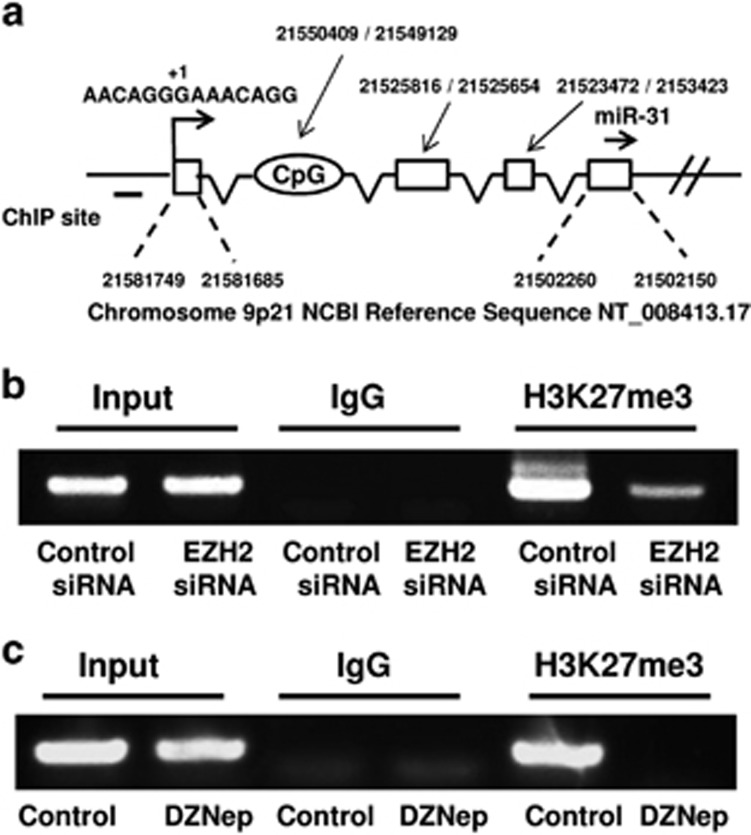
EZH2 regulates H3K27 methylation on the miR-31 promoter. (**a**) The putative transcription start site (indicated by +1) and genomic sequence of the miR-31 gene is shown. The mature miR-31 sequence is indicated by ‘→'. The DNA fragment covered by ChIP assay is indicated by ‘**−**.' The locations of the first four exons on the chromosomes were indicated. (**b**) PC-3 cells were transfected with the negative control or EZH2 targeting siRNAs for 48 h. ChIP assay was performed as described in Materials and Methods, using primers specific for the miR-31 promoter and the indicated antibodies. (**c**) PC-3 cells were treated with 5 *μ*M of DZNep for 24 h. ChIP assay was performed as in (**b**)

We performed 5′ rapid amplification of cDNA ends (5′ RACE) experiments to identify the transcription start site and the promoter for the miR-31 gene. The gene encoding miR-31 is located on chromosome 9p21. The transcription start site for miR-31 was identified (Figure 2a). To determine if EZH2 regulates histone H3K27 methylation, we performed chromosome immunoprecipitation (ChIP) assay near the transcription start site (~360 bp upstream) on the miR-31 promoter. As shown in Figures 2b and c, siRNA knockdown of EZH2 or DZNep treatment decreased the levels of H3K27 trimethylation. ChIP assay at 1250 bp upstream from the transcription start site did not detect H3K27 methylation, indicating that methylation occurs specifically at the transcription start site. As shown in Supplementary Figure 2, siRNA knockdown of EZH2 decreased the binding of EZH2 to the miR-31 promoter, whereas the levels of histone H3 on the promoter were not changed by EZH2 knockdown.

### Downregulation of EZH2 increases docetaxel-induced apoptosis in prostate cancer cells

**Figure 3:**
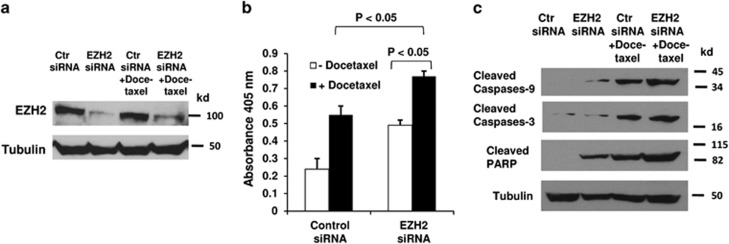
EZH2 knockdown increases docetaxel-induced apoptosis. PC-3 cells were transfected with the negative control or EZH2 targeting siRNAs. At 24 h after siRNA transfection, cells were treated with 10 nM docetaxel for additional 24 h. (**a**) Cell lysates were analyzed by western blot using the indicated antibodies. (**b**) Apoptosis was measured by Cell Death Detection Elisa^PLUS^ analysis as described in Materials and Methods. (**c**) Western blotting was performed with the indicated antibodies. The experiments have been repeated three times, and data shown are mean values±S.D.

As miR-31 regulates apoptosis by targeting the antiapoptotic protein E2F6,^25^ we hypothesized that by silencing miR-31, EZH2 may regulate apoptosis in prostate cancer cells. We transfected PC-3 cells with the negative control or EZH2 targeting siRNAs and treated the cells with docetaxel, a drug used clinically to treat prostate cancer. EZH2 knockdown increased miR-31 expression with or without docetaxel treatment (Supplementary Figure 3A). Docetaxel was able to induce higher level of apoptosis in cells that were transfected with EZH2 siRNA, compared with cells that were transfected with the negative control siRNA (Figure 3). Higher levels of active (cleaved) caspase-3 and caspase-9, as well as PARP cleavage, were observed in cells after EZH2 siRNA transfection and docetaxel treatment (Figure 3). In DU-145 cells, we also observed that siRNA knockdown of EZH2 increased miR-31 expression (Supplementary Figure 3B), docetaxel-induced apoptosis, and active caspase-3/caspase-9, as well as PARP cleavage (Supplementary Figures 1C and D).

### Overexpression of EZH2 confers resistance to docetaxel-induced apoptosis

**Figure 4:**
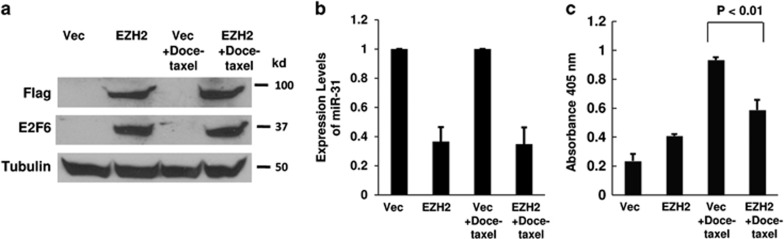
EZH2 confers resistance to docetaxel-induced apoptosis. PC-3 cells were transfected with the empty expression vector or pCEP4-EZH2. At 24 h after transfection, cells were treated with 10 nM of docetaxel for additional 24 h. (**a**) EZH2 and E2F6 expression was determined by western blot. (**b**) Total RNA was isolated from the cells and real-time PCR analysis was performed. (**c**) Apoptosis was measured by Cell Death Detection Elisa^PLUS^ analysis. The experiments have been repeated three times, and data shown are mean values±S.D.

We expressed EZH2 exogenously to determine if EZH2 can contribute to apoptosis resistance in prostate cancer cells. We transfected PC-3 cells with pCEP4-EZH2 expression vector to overexpress Flag-tagged EZH2 protein. Overexpression of EZH2 was confirmed by western blotting (Figure 4a). As expected, overexpression of EZH2 suppressed miR-31 (Figure 4b), and increased E2F6 protein (Figure 4a). When treated with docetaxel, cells expressing EZH2 were significantly more resistant to drug-induced apoptosis, compared with empty vector-transfected cells (Figure 4c).

### SiRNA knockdown of E2F6 sensitizes prostate cancer cells to docetaxel-induced apoptosis

**Figure 5:**
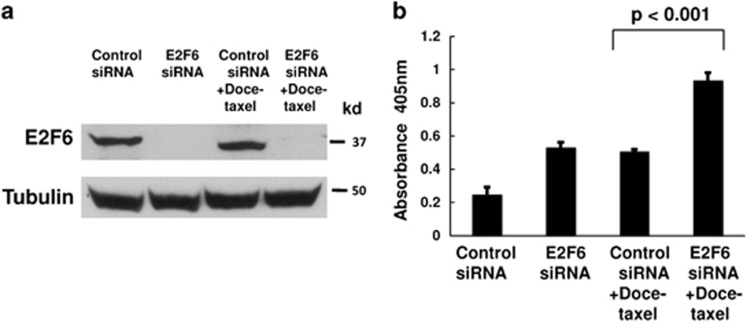
SiRNA knockdown of E2F6 sensitizes PC-3 cells to docetaxel-induced apoptosis. PC-3 cells were transfected with the negative control or E2F6 targeting siRNAs. At 24 h after siRNA transfection, cells were treated with 10 nM docetaxel for additional 24 h. (**a**) Cell lystates were analyzed by western blotting with the indicated antibodies. (**b**) Apoptosis was measured by Cell Death Detection Elisa^PLUS^ analysis. The experiments have been repeated three times, and data shown are mean values±S.D.

We have previously shown that miR-31 targets E2F6.^25^ E2F6 is an antiapoptotic protein that inhibits UV- and hypoxia-induced apoptosis.^28,29^ To determine the effects of E2F6 on chemotherapy-induced apoptosis in prostate cancer cells, we used siRNA to knockdown specifically E2F6 in PC-3 cells. As shown in Figure 5a, transfection of E2F6 targeting siRNA was able to decrease the level of E2F6 protein. SiRNA knockdown of E2F6 sensitized PC-3 cells to apoptosis induced by docetaxel (Figure 5b). In DU-145 cells, we also observed that siRNA knockdown of E2F6 increased docetaxel-induced apoptosis (Supplementary Figure 4). In both PC-3 and DU-145 cells, miR-31 levels were not affected by E2F6 knockdown or docetaxel treatment (Supplementary Figure 5).

### MiR-205 regulates miR-31 through EZH2

**Figure 6:**
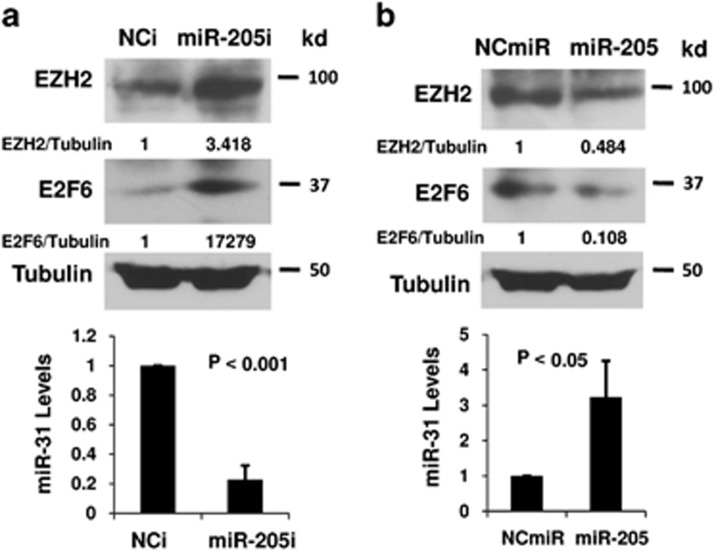
EZH2 integrates miR-205 silencing and miR-31 suppression. (**a**) WPE1-NA22 cells were transfected with negative control or anti-miR-205 inhibitor. After 48 h, total RNA and protein were collected and miR-31 expression was analyzed by real-time PCR and western blots were carried out using indicated antibodies. (**b**) PC-3 cells were transfected with the negative control miR or miR-205 for 48 h, and real-time PCR and western blots were performed as in (**a**). The experiments have been repeated three times, and data shown are mean values±S.D.

We have reported that miR-205 is silenced in prostate cancer by promoter hypermethylation.^25^ Interestingly, miR-205 has been shown to decrease EZH2 protein in prostate cancer cells.^30^ We hypothesized that the epigenetic silencing of miR-205 (through promoter methylation) will lead to increased expression of EZH2, which in turn epigenetically represses miR-31 expression through histone methylation. To test our hypothesis, we examined the effects of miR-205 on miR-31 expression. As shown in Figure 6a, blocking miR-205 with an anti-miR inhibitor in WPE1-NA22 cells (a cell line that expresses high level of endogenous miR-31^25^) decreased miR-31 expression, and increased EZH2 and E2F6 proteins. In contrast, overexpression of miR-205 in PC-3 cells caused a decrease of EZH2 and an increase of miR-31, which in turn decreased E2F6 (Figure 6b).

### MiR-205, EZH2, and miR-31 expression in human prostate cancer specimens

**Figure 7:**
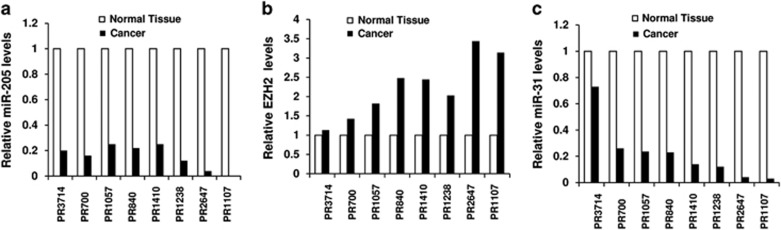
MiR-205, EZH2, and miR-31 expression in human prostate cancer specimens. Total RNAs were isolated from eight pairs of prostate cancer specimens and the adjacent normal prostate tissues. (Each pair of samples were from the same patient indicated by the patient ID on the X axis.) MiRNA and EZH2 expression were determined by real-time PCR. The ratios of miR-205 (**a**), EZH2 (**b**), and miR-31 (**c**) levels in the tumor samples *versus* that in the matched adjacent non-tumor samples were shown (the expression levels in each normal tissue were designated as 1)

We analyzed miR-205, EZH2, and miR-31 expression in eight pairs of human prostate cancer specimens and the adjacent non-malignant tissues using real-time PCR. We found that miR-205 and miR-31 expression levels were decreased in the cancer samples compared with the normal tissues (Figure 7). In the mean time, EZH2 expression was increased in the cancer specimens. Interestingly, the expression levels of miR-205, EZH2, and miR-31 correlate well among the individual patients (Pearson's correlation coefficient test: *R*=−0.7911 between the levels of EZH2 and miR-31; *R*=−0.6236 between EZH2 and miR-205). For example, low levels of miR-205 in patients PR2647 and PR1107 match to the high levels of EZH2 in the same patients, which in turn lead to the very low levels of miR-31 in these patients. This observation supports our hypothesis that EZH2 may coordinate the silencing of miR-205 and miR-31 in prostate cancer.

## Discussion

**Figure 8:**
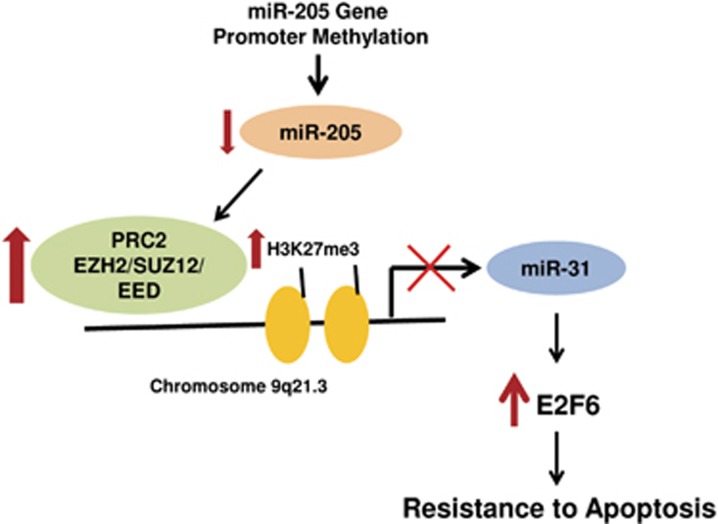
Schematic illustration of EZH2 coordination of miR-205 and miR-31 silencing in the development of apoptosis resistance. The promoter methylation-mediated silencing of miR-205 leads to the increase of EZH2 expression, which in turn suppresses miR-31 through H3K27 methylation on the miR-31 promoter. The reduction of miR-31 expression results in the increase of antiapoptotic protein E2F6, which confers resistance to apoptosis

Overexpression of EZH2 is found in various types of solid tumors such as melanoma,^31^ breast cancer,^6^ cervical cancer,^32^ gastric cancer,^33^ and prostate cancer.^4^ High levels of EZH2 are linked to tumor growth, metastasis, and poor prognosis for cancer patients. EZH2 suppresses apoptosis in a variety of cancers, including gastric cancer,^11^ bladder cancer,^12^ leukemia,^27^ and prostate cancer.^34^ However, the mechanisms of apoptosis suppression by EZH2 remain poorly understood. It has been reported that miR-205 suppresses EZH2 expression^30^ in prostate cancer cells and miR-205 promotes apoptosis.^25,35^ In this study, we identified a novel mechanism of EZH2 regulation of apoptosis in prostate cancer. As presented in a conceptual model (Figure 8), we propose that EZH2 coordinates the silencing of the proapoptotic miR-205 and miR-31. Thus, DNA methylation-mediated silencing of miR-205^25^ can lead to histone methylation-mediated silencing of miR-31, with EZH2 as the coordinator of the two separate events. As a result, the expression of antiapoptotic protein E2F6 is increased and contributes to the development of apoptosis resistance. It has been reported that the genomic loss of miR-101 can result in the upregulation of EZH2 in prostate cancer^36^ and miR-101 promote apoptosis in cancer cells.^12,37^ EZH2 may also contribute to apoptosis resistance by linking the genomic loss of miR-101 to miR-31 silencing.

At present, we do not know the mechanism of how miR-205 inhibits EZH2. There is no target sequence of miR-205 within the 3′-UTR of *EZH2* gene (http: //www.targetscan.org). Thus, miR-205 may suppress EZH2 indirectly through its action on the molecules that control EZH2 expression. For example, the oncogene Myc can regulate EZH2 at both transcriptional and posttranscriptional levels.^38^ Furthermore, overexpression of ERG (as a result of fusion between the TMPRESS2 and ERG from chromosome 21 translocation) can directly activate EZH2.^39^ Epigenetic silencing of miR-205 may result in the increase of these EZH2 activators, which in turn increases EZH2 expression.

Although a previous study has reported promoter methylation of the miR-31 gene,^40^ the methylation analysis was carried out in the region of the CpG island that is located in the intron region between exons 1 and 2 (Figure 2a). The CpG island is about 32 kb away from the transcription start site that we identified with 5′ RACE. Our ChIP assay was performed in the revised promoter region that has no CpG island (Figure 2), indicating that EZH2 may suppress miR-31 expression independent of DNA methylation. Previously, it has been shown that histone H3K27 trimethylation can silence the expression of miR-22 independent of promoter methylation.^41^

Various small-molecule drugs have been developed to target EZH2. For example, GSK126 is a very potent EZH2 inhibitor (with a *K*_i_ of 0.5–3 nM) and has high selectivity for EZH2 (more than 1000-fold higher activity than its activity for 20 other human methyltransferases).^42^ Importantly, GSK126 inhibits the growth of EZH2 mutant diffuse large B-cell lymphoma xenografts in mice.^42^ Our findings indicate that EZH2 functions as a key coordinator in apoptosis suppression. Targeting EZH2 with drugs like GSK126 may not only induce apoptosis by itself but also sensitize cancer cells to other agents of chemotherapy.

## Materials and Methods

### Cells and transfection

The cell lines WPE1-NA22, PC-3, and DU-145 were purchased from American Type Culture Collection (Manassas, VA, USA). The WPE1-NA22 cells were cultured in keratinocyte serum-free medium (Invitrogen, Grand Island, NY, USA), supplemented with bovine pituitary extract and human recombinant epidermal growth factor. PC-3 and DU-145 cells were cultured in RPMI-1640 media containing 10% FBS. For transient transfection, plasmids were transfected into cells using Lipofectamine Plus Reagent (Invitrogen) following the manufacturer's protocol. SiRNAs were transfected into cells using X-tremeGENE siRNA transfection reagent (Roche, Indianapolis, IN, USA) following the manufacturer's protocol.

### Drugs and chemicals

Docetaxel was purchased from Sigma (St. Louis, MO, USA) and DZNep was purchased from Cayman Chemical (Ann Arbor, MI, USA).

### Plasmids construction, siRNA, miRNA mimic, and inhibitor

The full-length *EZH2* cDNA was obtained by PCR using an EST clone as template and constructed into pCEP4 expression vector to express EZH2 as a Flag-tagged protein. SiRNAs targeting EZH2 (target sequence, 5′-GACUCUGAAUGCAGUUGCU-3′) and E2F6 (siRNA ID 4185) and negative control siRNA were purchased from Life Technologies (Grand Island, NY, USA). miRIDIAN miR-205 mimic (cat. no. C-300564-05) and negative control miRNA (cat. no. CN-001000-01-05) were purchased from Dharmacon (Lafayette, CO, USA). Anti-miR miRNA inhibitor for miR-205 (ID AM11015) was purchased from Life Technologies.

### Western blot analysis

Cells were lysed in RIPA buffer (1% NP-40, 0.5% sodium deoxycholate, 0.1% SDS in PBS). Complete protease inhibitor cocktail (Roche) was added to lysis buffer before use. Protein concentration was determined by Bio-Rad DC protein assay (Bio-Rad, Hercules, CA, USA). Protein samples were subjected to SDS-PAGE and transferred to nitrocellulose membrane. The membrane was blocked in 5% non-fat milk in PBS overnight and incubated with primary antibody and subsequently with appropriate horse radish peroxidase-conjugated secondary antibody. Signals were developed with ECL reagents (Pierce, Rockford, IL, USA) and exposure to X-ray films. Anti-β-tubulin and anti-E2F6 antibodies were purchased from Santa Cruz Biotechnology (Dallas, TX, USA). Cleaved caspase-9, cleaved caspase-3, cleaved PARP, and EZH2 antibodies were purchased from Cell Signaling (Danvers, MA, USA). Image digitization and quantification were carried out with UN-SCAN-IT software from Silk Scientific (Orem, UT, USA).

### Real-time PCR

Gene expression was measured by real-time PCR using TaqMan assays (cat. no. TM509 for miR-205, TM1100 for miR-31, Hs00544833_m1 for EZH2) from Applied Biosystems (Foster city, CA, USA). Total RNA was isolated using *mir*Vana miRNA Isolation Kit (Ambion, Grand Island, NY, USA). Five micrograms of total RNA was used in reverse transcription reaction. The cDNAs were used as templates to perform PCR on a Applied Biosystems 7500 Real-time PCR System (Applied Biosystems) following the manufacturer's protocol. Relative miRNA expression levels were calculated using 18S RNA as reference.

### 5′ RACE

The transcription start site of miR-31 pri-miRNA was identified by 5′ RACE experiments with FirstChoice RLM-RACE Kit from Ambion, using total RNA isolated from WPE1-NA22 cells as template.

### ChIP assay

ChIP assay was performed using the ChIP Assay Kit from Millipore (Billerica, MA, USA), following the supplied protocol. Immunoprecipitations were performed using anti-H3K27me3 (Active Motif) or control IgG antibodies. PCR was performed with the primers designed from the sequence of the miR-31 promoter (5′-GCTATCTCAACCCACTCTCCGCCT-3′ and 5′-GATTAGATGCTGATGTGAGTGCTG-3′), covering a ~200 bp fragment that is ~360 bp upstream of the transcription start site of the *miR-31* gene.

### Detection of apoptosis

The Cell Death Detection Elisa^PLUS^ Kit (Roche) was used to detect apoptosis following the manufacturer's protocol. This assay determines apoptosis by measuring mono- and oligonucleosomes in the lysates of apoptotic cells. The cell lysates were placed into a streptavidin-coated microplate and incubated with a mixture of anti-histone-biotin and anti-DNA-peroxidase. The amount of peroxidase retained in the immunocomplex was photometrically determined with ABTS as the substrate. Absorbance was measured at 405 nm.

### Human prostate cancer specimens

Frozen tissues of human prostate cancer specimens and paired normal prostate tissues were obtained from Mayo Clinic SPORE, and approved by the Mayo Clinic Institutional Review Board. These patients had biopsy-proven prostate cancer and were treated at the Mayo Clinic by radical retropubic prostatectomy without neoadjuvant therapy.^43^

### Statistical analysis

Differences between the mean values were analyzed for significance using the unpaired two-tailed Student's *t*-test for independent samples; *P*≤0.05 was considered to be statistically significant. Correlation significance was assessed using Pearson's correlation coefficient test.

## Abbreviations

miRNA: microRNA
EZH2: enhancer of zeste homolog 2
E2F6: E2F transcription factor 6
ChIP: chromatin immunoprecipitation
5′ RACE: 5′ rapid amplification of cDNA ends
