# Supplementary material for: Polycomb protein EZH2 suppresses apoptosis by silencing the proapoptotic miR-31
Source: Cell Death Dis. 2014 Oct 23;5(10):e1486–. doi: 10.1038/cddis.2014.454 (PMC4237267; doi:10.1038/cddis.2014.454)
Supplement: Supplementary Material [file cddis2014454x1.pdf]

Zhang et al, 2014 Cell Death and Disease Manuscript

Supplemental Materials

1. Supplemental Figure Legend

Supplemental Figure 1 EZH2 suppresses miR-31 expression. (A and B) Left, DU-145 cells were transfected with the negative control or EZH2 targeting siRNAs for 48h. Right, DU-145 cells were treated with 5 μM of DZNep for 24 hours. (A) Cell lysates were analyzed by western blot using the indicated antibodies. (B) Total RNA was isolated from the cells and real-time PCR analysis was performed as described in Materials and Methods. (C and D) DU-145 cells were transfected with the negative control or EZH2 targeting siRNAs. At 24h after siRNA transfection, cells were treated with 10 nM docetaxel for additional 24h. (C) Apoptosis was measured by Cell Death Detection Elisa^{PLUS} analysis as described in Materials and Methods. (D) Western blotting was performed with the indicated antibodies. The experiments have been repeated three times, data shown are mean values + SD.

Supplemental Figure 2 PC-3 cells were transfected with the negative control or EZH2 targeting siRNAs for 48h. ChIP assay was performed as described in Materials and Methods, using primers specific for the miR-31 promoter and the indicated antibodies. The experiments have been repeated three times.

Supplemental Figure 3 PC-3 cells (A) and DU-145 cells (B) were transfected with the negative control or EZH2 targeting siRNAs. At 24h after siRNA transfection, cells were treated with 10 nM docetaxel for additional 24h. Total RNA was isolated from the cells and real-time PCR analysis was performed as described in Materials and Methods. The experiments have been repeated three times, data shown are mean values + SD.

Supplemental Figure 4 DU-145 cells were transfected with the negative control or E2F6 targeting siRNAs. At 24h after siRNA transfection, cells were treated with 10 nM docetaxel for additional 24h. (A) Cell lystates were analyzed by western blotting with the indicated antibodies. (B) Apoptosis was measured by Cell Death Detection ElisaPLUS analysis. The experiments have been repeated three times, data shown are mean values + SD.

Supplemental Figure 5 PC-3 cells (A) and DU-145 cells (B) were transfected with the negative control or E2F6 targeting siRNAs. At 24h after siRNA transfection, cells were treated with 10 nM docetaxel for additional 24h. Total RNA was isolated from the cells and real-time PCR analysis was performed as described in Materials and Methods. The experiments have been repeated three times, data shown are mean values + SD.

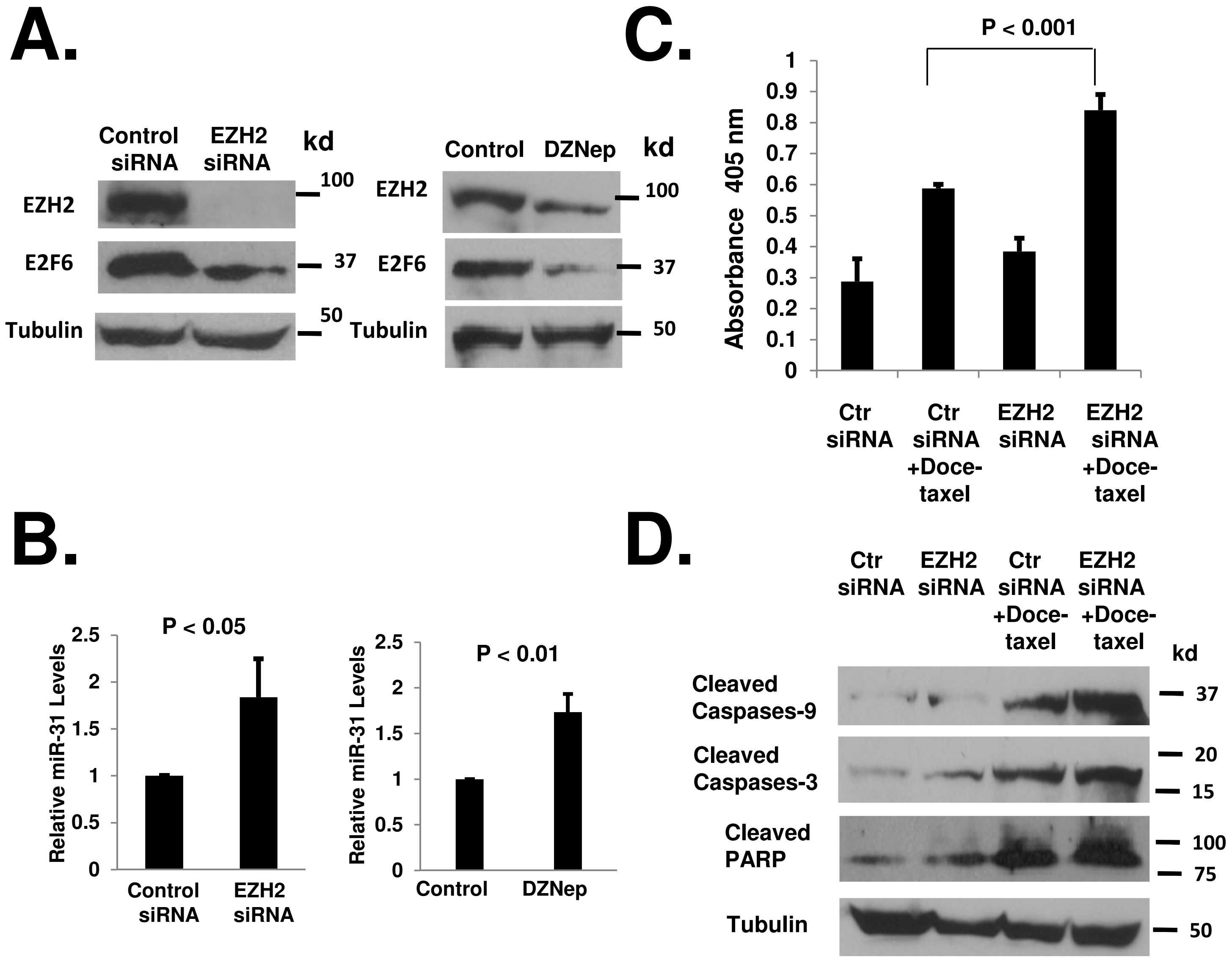

Supplemental Figure 1

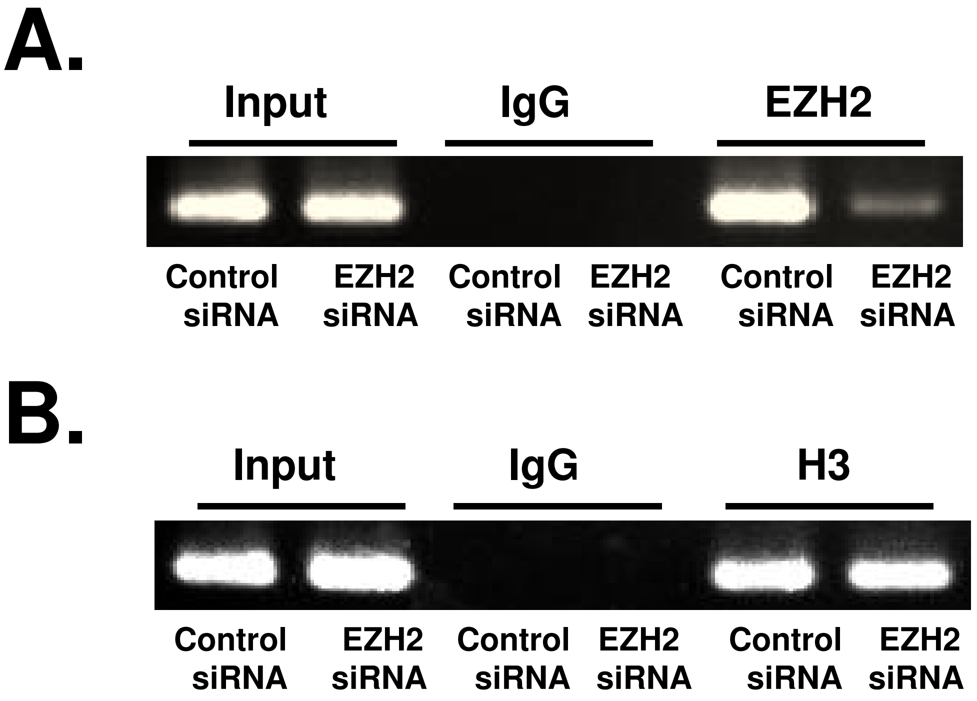

Supplemental Figure 2

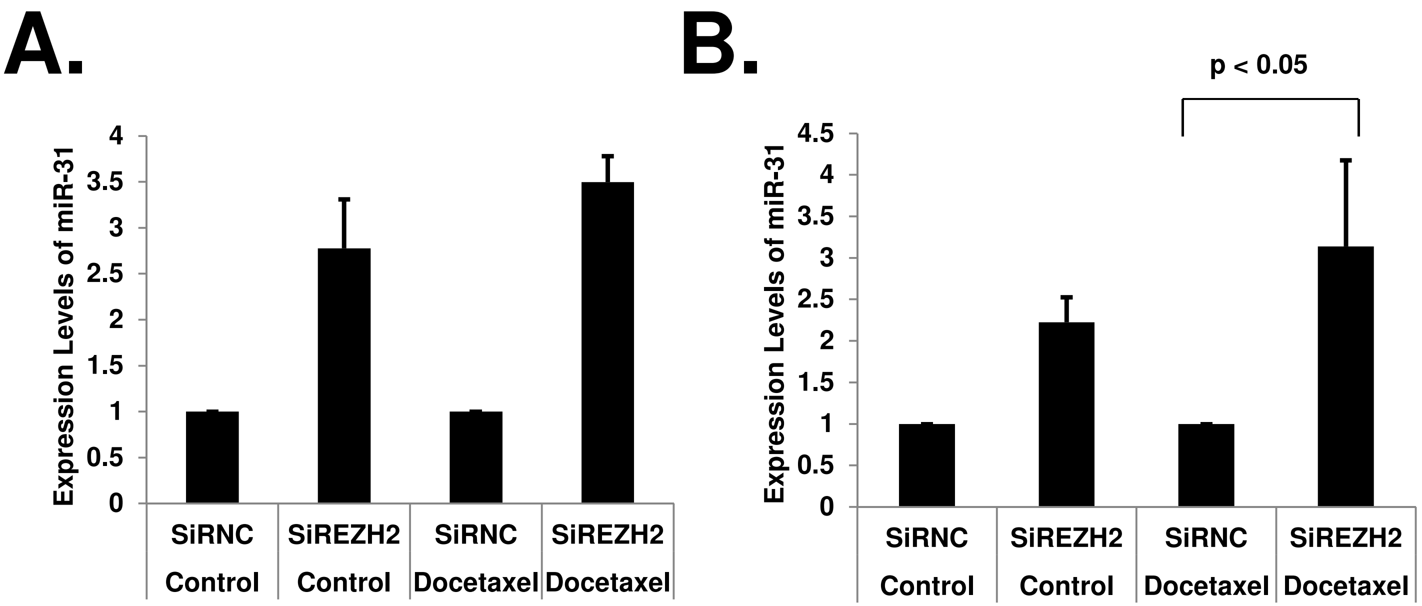

Supplemental Figure 3

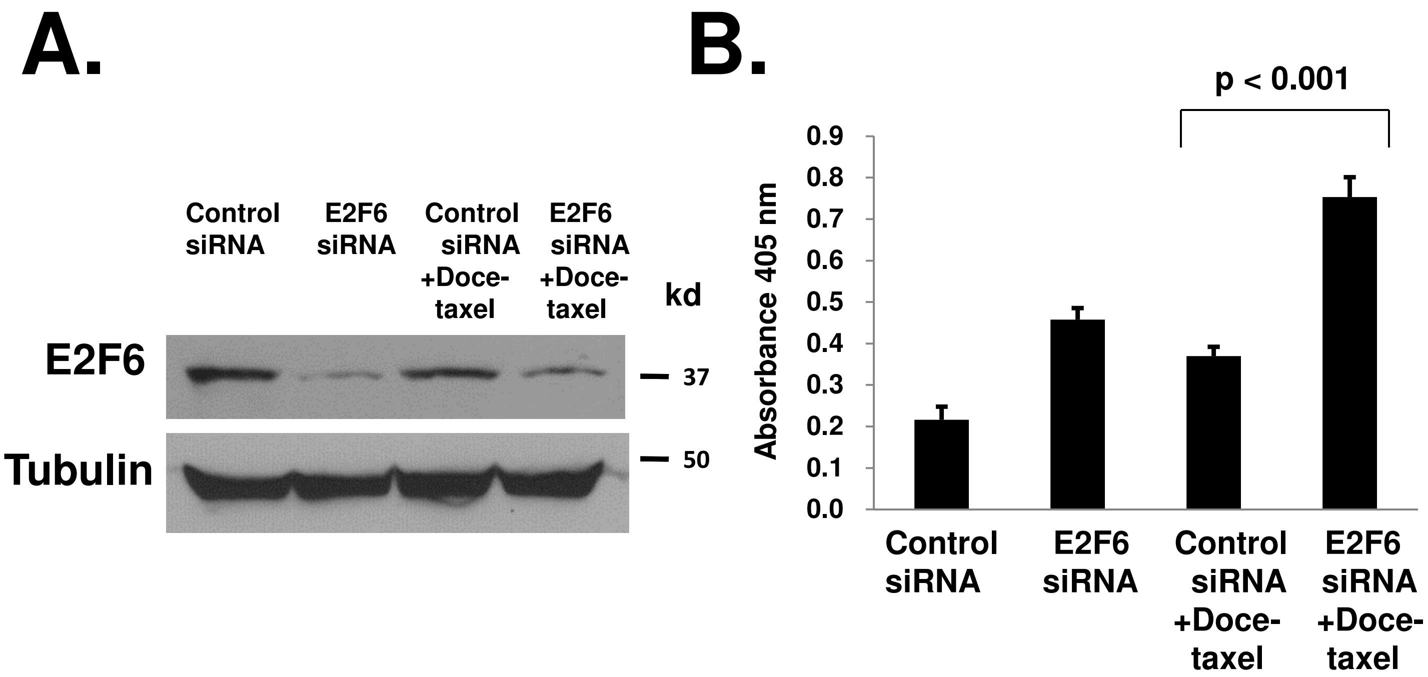

Supplemental Figure 4

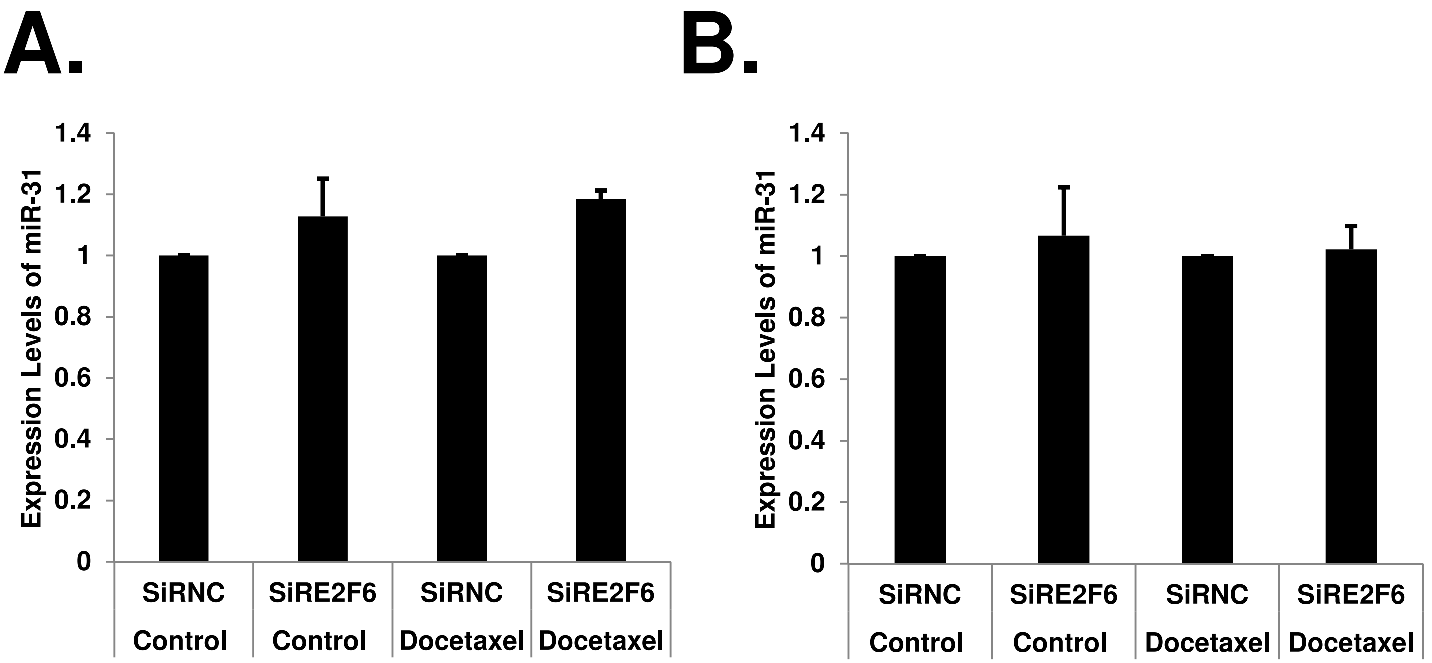

Supplemental Figure 5
